# Current and Potential Uses of Immunocytokines as Cancer Immunotherapy

**DOI:** 10.3390/antib1020149

**Published:** 2012-07-04

**Authors:** Paul M. Sondel, Stephen D. Gillies

**Affiliations:** 1 The Departments of Pediatrics, Human Oncology, and Genetics and The UW Carbone Cancer Center, University of Wisconsin, Madison WI; 2 Provenance Biopharmaceuticals Corp. Burlington MA

**Keywords:** immunocytokine, ADCC, cancer, immunotherapy

## Abstract

Immunocytokines (ICs) are a class of molecules created by linking tumor-reactive monoclonal antibodies to cytokines that are able to activate immune cells. Tumor selective localization is provided by the ability of the mAb component to bind to molecules found on the tumor cell surface or molecules found selectively in the tumor microenvronment. In this way the cytokine component of the immunocytokine is selectively localized to sites of tumor and can activate immune cells with appropriate receptors for the cytokine. Immunocytokines have been made and tested by us, and others, using a variety of tumor-reactive mAbs linked to distinct cytokines. To date, the majority of clinical progress has been made with ICs that have linked human interleukin-2 (IL2) to a select number of tumor reactive mAbs that had already been in prior clinical testing as non-modified mAbs (Figure 1). Here we briefly review the background for the creation of ICs, summarize current clinical progress, emphasize mechanisms of action for ICs that are distinct from those of their constituent components, and present some directions for future development and testing.

## 1. Cancer Immunotherapy: Broad Application

Despite over 50 years of compelling preclinical evidence for effective cancer immunotherapy, as of 2009 there were only 4 widely used, approved, effective clinical immunotherapies being used broadly to treat cancer. They included: (A) allogeneic bone marrow transplantation for leukemia, largely successful via the “graft versus leukemia” effect [1]; (B) tumor reactive monoclonal antibodies (mAbs), effective in part via antibody dependent cell-mediated cytotoxicity (ADCC) [2]; (C) intravesical BCG for superficial bladder cancer, likely active via toll-like receptors on leukocytes [3]; and (D) administration of IL2 for melanoma and renal cell cancer [4].

Over the past 2 years Phase III trials of 3 distinct forms of immunotherapy demonstrated clinical benefit. Each was published in the New England Journal of Medicine. Two are now FDA approved and the 3rd is under FDA review. They are: (1) Vaccination of patients (pts) with advanced prostate cancer, with a preparation of PAP-loaded autologous dendritic cells [5]; (2) Augmenting endogenous anti-melanoma immunity via anti-CTLA-4 mAb [6,7]; and (3) Enhancing ADCC in children with neuroblastoma (NBL) by combining an anti-GD2 mAb with NK activation (via IL2) and neutrophil/monocyte activation (via GM-CSF) [8].

The use of ICs for cancer treatment is, at least in part, based on augmenting ADCC by the more effective localization of cytokines able to activate ADCC.

## 2. ADCC and FcRs

Preclinical studies have shown that tumor-reactive mAbs can mediate *in vitro* tumor destruction via ADCC [9,10]. While there is a family of FcRs on leukocytes [11], the most important for ADCC are the FcRγ2a (CD32) FcRs expressed primarily on neutrophils, monocytes and macrophages, and the FcRγ3a (CD16) FcRs expressed primarily on NK cells. The affinity of these human FcRs is highest for human IgG1 immunoglobulins [12]. When a sufficient concentration of a tumor-reactive mAb encounters a tumor cell (e.g., Rituximab and a CD20^+^ tumor cell), the mAb binds to the cell, presenting a lattice of surface bound mAb molecules with exposed Fc epitopes. When an effector expressing FcRs (such as an NK cell) encounters this mAb-coated tumor cell, the FcRs simultaneously engage multiple Fc epitopes via multipoint binding. The multipoint binding enables the effector cell to transiently adhere to the mAb-coated tumor cell (akin to the multipoint adhesion facilitated by “a Velcro effect”), and then to activate the effector cell via the signaling pathways induced by the FcRs [13]. This mAb-FcR mediated activation of the tumor-bound effector cell can result in cytokine and chemokine release by the effector cell, antigen ingestion/presentation, and “downstream” immune recruitment and activation of other antitumor effector cells [14]. In addition, this mAb-FcR mediated activation of the tumor-bound effector cell can result in activation of ADCC, which may involve granule induced cell death, death-signal induced apoptosis, or biochemical induced toxicity, depending upon the effector cell type and state of activation [15,16]. For simplicity, we will refer to these pathways (direct ADCC and FcR induced “downstream” effects) as “*in vivo* ADCC”. In mice, antitumor effects induced by mAbs that mediate ADCC are abrogated when using mAbs without functional Fc components [9], or in mice lacking functional FcRs [17].

The importance of FcR affinity in the *in vivo* efficacy of mAbs has been demonstrated in several clinical studies. The human CD16 FcRγ3a molecule has 2 primary alleles; a lower affinity allele that bears a phenylalanine (F) at amino acid (a.a.) 158, and a higher affinity allele that bears a valine (V) at a.a. 158. Analogously, the human CD32 FcRγ2a molecule also has 2 primary alleles; a lower affinity allele that bears an arginine (R) at a.a. 131, and a higher affinity allele that bears a histidine (H) at a.a. 131. Landmark studies demonstrated that lymphoma patients with high affinity FcR alleles were more likely to benefit from Rituximab than patients without high affinity FcRs [18,19]. Furthermore, the presence or absence of complement inactivators [20] (such as CD59) on lymphomas does not seem to influence Rituximab efficacy [21]. The CD16 FcRγ3a allelic polymorphism affects rituximab- mediated ADCC of autologous EBV transformed B lymphocytes *in vitro* [22]. As expected, this cytotoxicity is attenuated by inhibitory killer-immunoglobulin-like receptors (KIR) found primarily on NK cells, but to a variable degree in different individuals [22]. Together, these observations indicate that the *in vivo* efficacy of Rituximab is likely dependent on *in vivo* ADCC rather than complement activation [23] and that the degree of cytotoxicity may also be regulated by KIR.

Herceptin and Erbitux are FDA-approved anti-tumor-receptor (HER2 and EGFR) mAbs that inhibit the natural ligands for these receptors from stimulating tumor cell growth [24]. In addition, both these mAbs mediate ADCC *in vitro*. Clinical studies for these mAbs have demonstrated greater benefit in individuals with high affinity alleles for FcRγ2a and FcRγ3a [25,26], analogous to that seen for Rituximab. These results suggest that at least some of the antitumor benefits of Herceptin and Erbitux come from *in vivo* ADCC, although increased signal inhibition and apoptosis resulting from the cross- linking of cell-bound antibody by FcR-expressing cells cannot be ruled out [27]. While not all analyses of Rituximab, Herceptin and Erbitux have shown identical results, most have demonstrated benefit for individuals with high affinity FcR alleles [28]. In addition, there is still controversy regarding the “gene dose” effect for these alleles. Namely the high affinity genotypes (VV for FcRγ3a and HH for FcRγ2a) show better antitumor effects than the lowest affinity genotypes (FF and RR respectively). However it is not clear whether the heterozygotes (*i.e*., VF and HR) show effects that are similar to the low affinity homozygotes (FF and RR), or potentially function somewhere in between the homozygotes (*i.e*., VF functioning between VV and FF, and HR functioning between HH and RR) [26].

In addition, FcR affinity influences the antitumor efficacy of antitumor antibodies induced by vaccination regimens. Having high affinity FcRγ2a and FcRγ3a genotypes is associated with beneficial antitumor effects for immunization to idiotypic antigens on B cell tumors [29] and to antigens on colon cancer [30].

## 3. Augmenting ADCC with Effector Activation, Ch 14.18 + cytokines

IL2 is a potent activator of NK cells [31,32], which have FcRs and mediate ADCC [13]. *In vitro* treatment of NK cells with IL2 augments NK ADCC [33]. We demonstrated that *in vivo* administration of IL2 to patients augmented the ability of their circulating NK cells to mediate ADCC *in vitro* [34]. We proposed that activating NK cells *in vivo* with IL2 would thus enhance *in vivo* ADCC from concomitant mAb treatment [35,36]. Our studies utilized mAbs that recognize the GD2 disialoganglioside on melanoma and NBL [37]. Our preclinical data suggested efficacy would be most apparent when this approach was used to treat individuals with smaller amounts of cancer (non-bulky disease) [38,39]. We performed a series of pilot and phase I/II trials of this approach for patients with neuroblastoma (NBL) or melanoma using the 14.G2a murine antibody and its derivative, the ch14.18 chimeric mAb [40–42]. We worked with the Children’s Oncology Group (COG) to test this approach clinically in the minimal disease setting in a pilot COG Phase I trial for children with high-risk NBL that were in remission after autologous HSCT (ASCT) but likely to relapse [43]. To augment ADCC we incorporated IL2 to activate NK cells and GM-CSF to activate neutrophils/macrophages [44–46]. The regimen was tolerated acceptably, and clinical results appeared better than historical controls [43]. We then moved this same regimen into a large Children’s Oncology Group (COG) phase III trial [8]. With only 61% of anticipated accrual, our biostatisticians stopped and “unblinded” the study, since the immunotherapy treatment was statistically superior to the control treatment for both event-free survival (66% *vs*. 46% *p* = 0.01), and for overall survival (86% *vs*. 75% *p* = 0.02). A separate German study of this same ch14.18 mAb, in a similar dose and regimen, but without the use of IL2 and GM-CSF initially reported no immunotherapeutic advantage for the ch14.18 mAb [47]. This suggests, although does not prove, that adding the IL2 and GM-CSF (to augment ADCC) to the ch14.18 treatment was responsible for the clinical benefit. Furthermore, these data from this COG study [8] suggest that other ADCC-inducing mAbs (*i.e.*, Rituximab, Herceptin and Erbitux) might be considered for trials in which high risk patients likely to relapse receive these mAbs in combination with agents known to activate ADCC (like IL2 + GM-CSF).

## 4. Immunocytokines: Linking IL2 to anti-GD2 mAb; Preclinical Development

**Figure 1:**
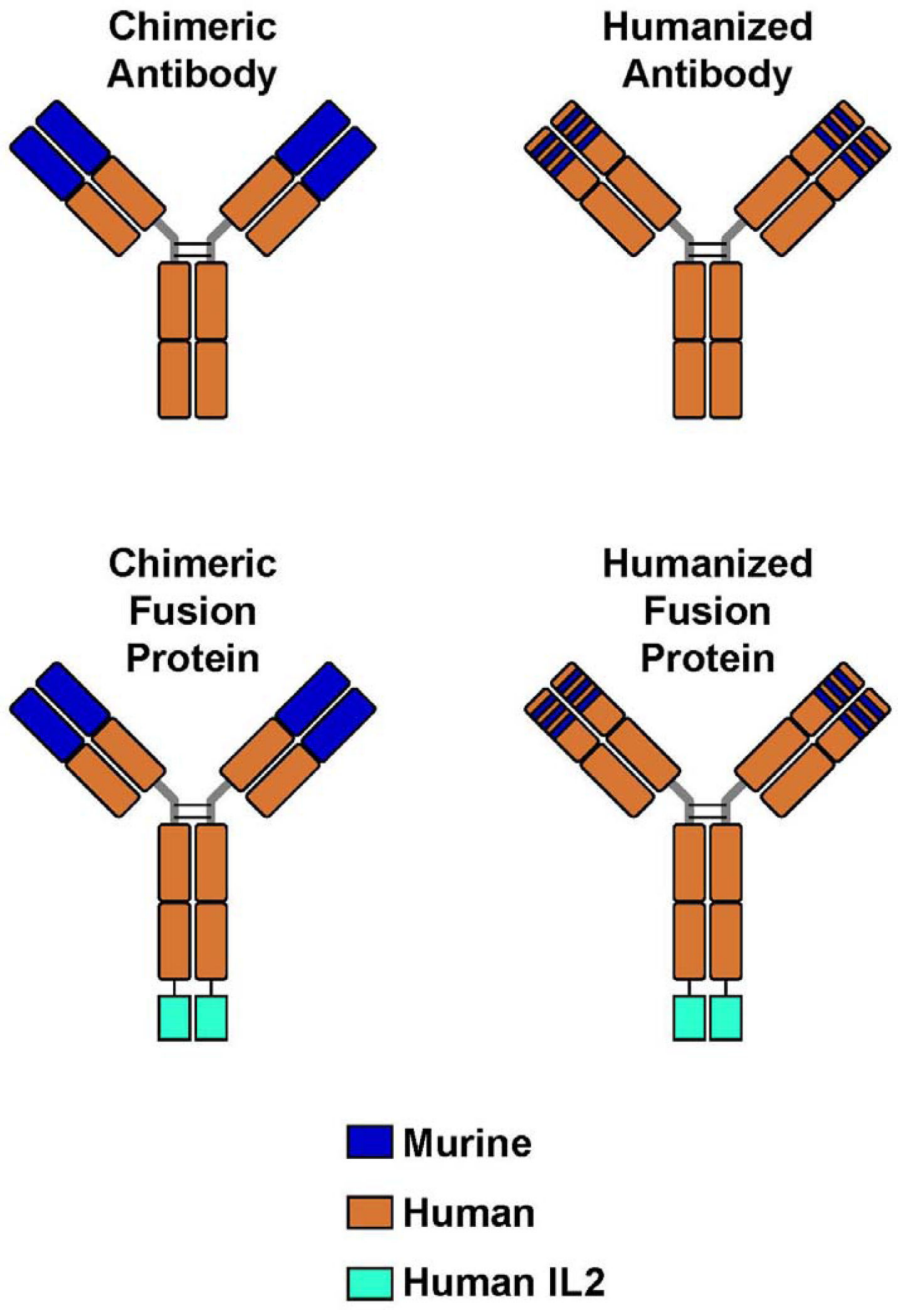
Monoclonal Antibodies and Immunocytokines. (**A**) A chimeric monoclonal antibody (mAb) combines the constant region of a human antibody with the variable domain of a murine antibody. The antigen specificity is conferred by the murine variable domain. (**B**) In the humanized mAb, the murine framework determinants of both the heavy and light chains are replaced with human framework determinants, but the antigen specificity of the original murine mAb is retained. (**C**, **D**) Immunocytokines combine the mAb with covalently linked cytokines, such as molecules of interleukin 2 (IL-2), in this case to the end of each of the heavy chains at the C-terminus.

Despite the efficacy of anti-GD2 mAb + cytokines in our recent NBL trial (Figure 1) [8], only 66% are NBL-free at 2 years. We aim to further enhance the clinical potency of ADCC, in order to obtain even better clinical results. Preclinical data with the hu14.18-IL2 IC indicate that this should be possible. ADCC depends, in part, upon the number and function of FcRs on the effector cells [25–30]. When NK cells are stimulated with IL2 *in vivo,* they mediate augmented ADCC [34]. However, we have shown that up to 50% of the activated NK cells circulating in cancer patients following *in vivo* treatment with IL2 do not have FcRs, in contrast to most resting NK cells [48]. These FcR^−^ activated NK cells are more lytic to tumor cells in direct assays not dependent on mAb and FcRs. We also found that NK cells activated *in vivo* by IL2 show augmented expression of the IL2Rβ [49] and enhanced *in vitro* responses to IL2 [50]. Furthermore, IL2R-bearing T cells that may not be able to specifically recognize tumors (with their TCRs) should still be responsive to IL2. It may be beneficial to activate these IL2R^+^ effector cells with a molecule that bridges them to tumor cells and then activates them. These are some of the functions of the anti-GD2 IC hu14.18-IL2 (Figure 1) and its preclinical predecessor ch14.18-IL2. These ICs were constructed by fusing the human IL2 gene to the ch14.18 or hu14.18 IgG1 genes [51]. The Gillies, Reisfeld, and Sondel labs have shown that these ICs activate GD2-specific tumor cell lysis by IL2R^+^ T cells and NK cells [51,52]. Ch14.18-IL2 induces anti-melanoma activity in a SCID –xenograft model [53] and in conventional mice bearing syngeneic tumors expressing GD2 (B78 melanoma) [54,55], and anti-NBL activity in conventional mice bearing the GD2^+^ NXS2 NBL [56,57].

**Figure 2:**
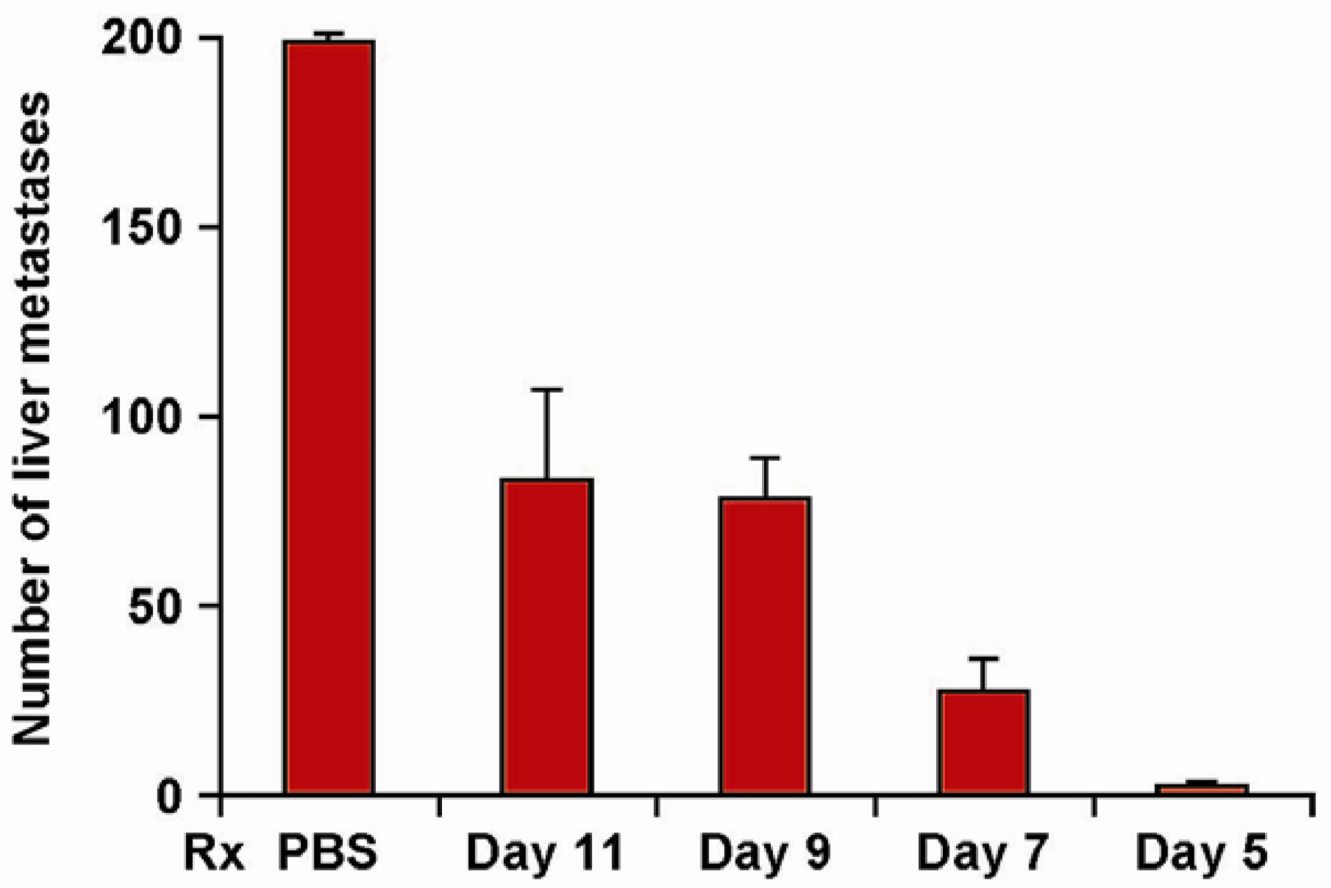
Efficacy of hu14.18-IL2 therapy is influenced by the timing of its initiation. Groups of 4 mice were treated IV with 5µg/d X 5d of hu14.18-IL2 beginning on d 5, 7, 9 or 11 following IV injection of 5 × 10^5^ NXS2 NB cells (or IV PBS). Mice were sacrificed on d28 and the liver metastases were enumerated. Data are mean ± SE (adapted from Neal et al, 2004 [39]).

Ch14.18-IL2 IC causes dramatically better antitumor effects against localized or metastatic NXS2 NBL than comparable amounts of ch14.18 mAb and IL2 in combination (Table 1) [56,57]. Under these conditions, IC-treated mice show no metastases. The *in vivo* destruction of NXS2 in mice receiving ch14.18-IL2 is largely NK mediated [56,57], while the antitumor effect against the B78 melanoma involves T cells [54]. This T cell effect demonstrates epitope spread, as ch14.18-IL2 enables C57Bl/6 mice to destroy GD2^−^ B16 melanoma cells, but only if they are a component of mixed tumors, created by co-injection with the GD2^+^ B78 melanoma cells (Table 1) [55]. Thus ch14.18-IL2 induces far more potent antitumor effects in melanoma or NBL-bearing mice than the combination of ch14.18 mAb + IL2, and functions both as a T cell inducing vaccine and a potent activator of NK- mediated ADCC. More potent tumor eradication is seen in mice with smaller tumor burdens (Figure 2) [39]. Similar results are seen in murine models using the hu14.18-IL2 IC [58]. As a nearly “pure” human protein it is predicted to be less immunogenic in patients than would ch14.18-IL2.

### 4.1. Phase I Clinical Testing of hu14.18-IL2

These results supported the FDA-IND for our “first in human” Phase I study in adults with melanoma at the UWCCC [59], and enabled our team to conduct a Phase I trial of hu14.18-IL2 in children [60], using the same schedule used in our melanoma trial.

Our Phase I trials in melanoma [59], and NBL [60], identified the MTD and immune effects of 3 daily IV doses of hu14.18-IL2. Of the 28 patients with measurable NBL, 3 showed isolated marrow improvement, and 1 showed a non-confirmed CR that was not completely attributable to the IC. Of the 28 melanoma patients with measurable disease, none showed a response. Of the 5 melanoma patients that entered with no evidence of disease (NED) following recent surgical resection of progressive metastases, 3 patients recurred at 1, 6 and 92 months, and 2 remain in remission > 74 and >117 months following treatment. Based on the suggestion of IC activity in melanoma patients with NED, the BM-activity seen in our NBL study, and our preclinical murine data documenting better antitumor efficacy for smaller tumors (Figure 2) [39], we proposed that greater antitumor activity would be detected when using hu14.18-IL2 to treat patients with less “bulky” tumors.

### 4.2. Phase II Clinical Testing of hu14.18-IL2

We thus worked with the COG to conduct A Phase II study of hu14.18-IL2 in Children with Recurrent or Refractory NBL. This Phase II protocol was designed to evaluate the clinical antitumor activity and *in vivo* biological-immunological effects of hu14.18-IL2, in children with refractory or recurrent NBL, and separately assess patients with bulky disease and patients with minimal evaluable NBL. Patients received 3 daily IV doses of 12.0 mg/M^2^/d IC in each of 4 monthly courses. Patients with CR or PR could receive 2 more courses [61].

Fifteen patients had disease measurable by standard radiographical criteria (stratum 1) and 24 patients had disease evaluable only by MIBG and/or BM histology (stratum 2). Responses were confirmed by independent radiological review and immunocytochemistry (ICC) evaluation of BM. No responses were seen in the 15 stratum 1 pts. In the 24 stratum 2 pts, 5 showed CR (complete resolution of MIBG avid disease and complete resolution of BM disease both by standard morphology and ICC). Of these 5 pts, 4 relapsed after 8, 12, 18 and 30 months, while 1 remains in CR after 35^+^ mo. At study entry, all 5 had recurrent-refractory disease following ASCT [61].

Two other stratum-2 patients showed disease improvement suggesting efficacy, but did not quite meet protocol criteria for PR or CR. One pt had BM-CR and a decrease in MIBG-avid disease that was read as a PR by the treating center but not by independent review. A 2nd pt had resolution of MIBG^+^ disease and BM improvement, but not clearing.

The spectrum and degree of toxicities were similar to that seen in our Phase I studies. Of the 38 patients evaluable for toxicity, 10 did not receive cycle 2 of therapy [2 patients due to protocol defined DLT, 7 because of progressive disease (PD) and 1 due to parental choice]. Of the 28 patients receiving cycle 2, 3 patients had toxicity in cycle 1 (pain, hypotension and hyperbilirubinemia) requiring a reduction in dose for cycle 2 [61].

The clinical response data support the conclusion that this agent and regimen have clinical activity in stratum-2 but not in stratum-1 pts. Although this study was not powered to address whether response may be predicted by entry status, identifying 5 CRs and 2 patients with clear improvement in the 24 patients in stratum-2 *vs*. 0 responses of 15 patients in stratum-1 suggests there may be a real difference (*p* = 0.03). As all patients in this study had recurrent/refractory disease following extremely aggressive prior multi-modality therapy, these responses are of interest to pediatric oncologists. COG is currently performing a follow-up feasibility-Phase II trial with this IC in combination with GM-CSF and cis-retinoic acid, focused on high risk NBL patients with non-bulky (stratum-2) disease to confirm/extend these response data to potentially support licensing of this agent. Furthermore, in order to evaluate potential mechanisms of anti-tumor response, we investigated whether the inhibitory NK Killer Immunoglobulin-like receptors (KIR) may be involved in regulating this clinical antitumor activity in patients receiving hu14.18-IL2.

## 5. The Roles of KIR/KIR-Ligand (KIR-L) and FcR Genotypes in the Responses Induced by hu14.18-IL2

The Killer Immunoglobulin-like Receptor (KIR) genes encode receptors that recognize MHC class I molecules. On a cellular level the expression of KIR and HLA defines the repertoire and responsiveness of NK cells. While there are both activating and inhibitory KIR gene products, most (but not all) clinical attention has been focused on the inhibitory KIR molecules. The inhibitory KIR receptors transmit inhibitory signals to NK cells when they encounter their cognate MHC class I molecules [62–70]. The ligand specificity is focused on amino acid position 80 of the HLA class I. As presented in Table 1, HLA-C alleles with Lys80 constitute ligands for KIR2DL1 and those with Asp80 interact with KIR2DL2 and KIR2DL3. Exceptions to this rule have recently been reported [129,13071,72]. KIR3DL1 recognizes HLA-B alleles with the so-called Bw4 motif, also conferred by amino acid positions 77–80. As recently shown, HLA-A alleles with the Bw4 motif can serve as ligands for KIR3DL1 [73]. Ruggeri et al. first reported on the phenomenon of KIR-L incompatibility and response to HLA-haploidentical HSCT, primarily in adult patients with acute myeloid leukemia (AML) [74,75]. According to this analysis, improved leukemia control is seen when there is a difference in HLA between the donor and recipient such that the recipient’s cells lack the ligand specific for the donor KIR, creating a “missing KIR ligand” situation. Leung et al. proposed the principle of missing KIR ligand analysis (designated here as KIR/KIR-L mismatch), in which the HSCT recipient lacks one or greater HLA class-I ligands for the HSCT donor’s inhibitory KIRs (regardless of whether or not the donor’s own HLA provides such ligands) [76]. They found that the response of pediatric patients with AML and acute lymphoid leukemia (ALL) to haploidentical HSCT could be predicted by the presence of this KIR/KIR-ligand mismatch. The KIR/KIR-L mismatch principle also posits that a difference in HLA between the donor and recipient is not necessary for the benefit of KIR-HLA mismatching. This was confirmed when an analysis of results of HLA-identical T cell depleted sibling HSCT also revealed a benefit of KIR/KIR-ligand mismatch [77].

The genes encoding for KIR and HLA class I KIR ligands are polymorphic and inherited independently [78]. As such, individuals differ with respect to the number of KIR genes present in the genome and frequently express KIR receptors that have no corresponding HLA ligands on autologous cells, thus displaying an “autologous” KIR-KIR ligand mismatch. This scenario of autologous KIR/KIR-L mismatch occurs in approximately 60% of the US population and has been implicated as a favorable prognostic factor in pediatric solid tumor patients following ASCT [78,79].

Until 2010 the analyses of KIR/KIR-L mismatch in the setting of cancer treatment had been confined to the clinical setting of allogeneic HSCT [70,71,77], allogeneic adoptive NK infusions [80–82] and autologous HSCT [78,79]. We hypothesized that this KIR/KIR-L relationship pertains more to the cellular function of NK cells, independent of allogeneic relationships [83] or autologous HSCT. We also hypothesized that individuals that were KIR/KIR-L mismatched would be more likely to respond favorably to cancer immunotherapy that is NK-mediated. Our preclinical data with the hu14.18-IL2 IC demonstrated that antitumor effects in mice could be mediated by NK cells, in the absence of T cells [39]. Furthermore, we showed that murine NBLs that escape from suboptimal hu14.18-IL2 regimens do so by up-regulating MHC class I, a mechanism that apparently turns off NK cells via the MHC-specific inhibitory Ly 49 receptors on murine NK cells [58]. These observations suggested that circumventing KIR mediated inhibition of NK cells may be helpful in the *in vivo* efficacy of this form of immunotherapy. Thus we hypothesized that individuals that were “autologous KIR/KIR-L mismatched” would have better NK function against their autologous tumor when treated with the hu14.18-IL2 reagent. Our phase II trial of hu14.18-IL2 treated 38 pts [61]. Five patients showed CR and 2 showed clear clinical benefit, that did not quite meet PR/CR criteria [61]. This provided the opportunity to look at the role of KIR/KIR-L.

**Figure 3:**
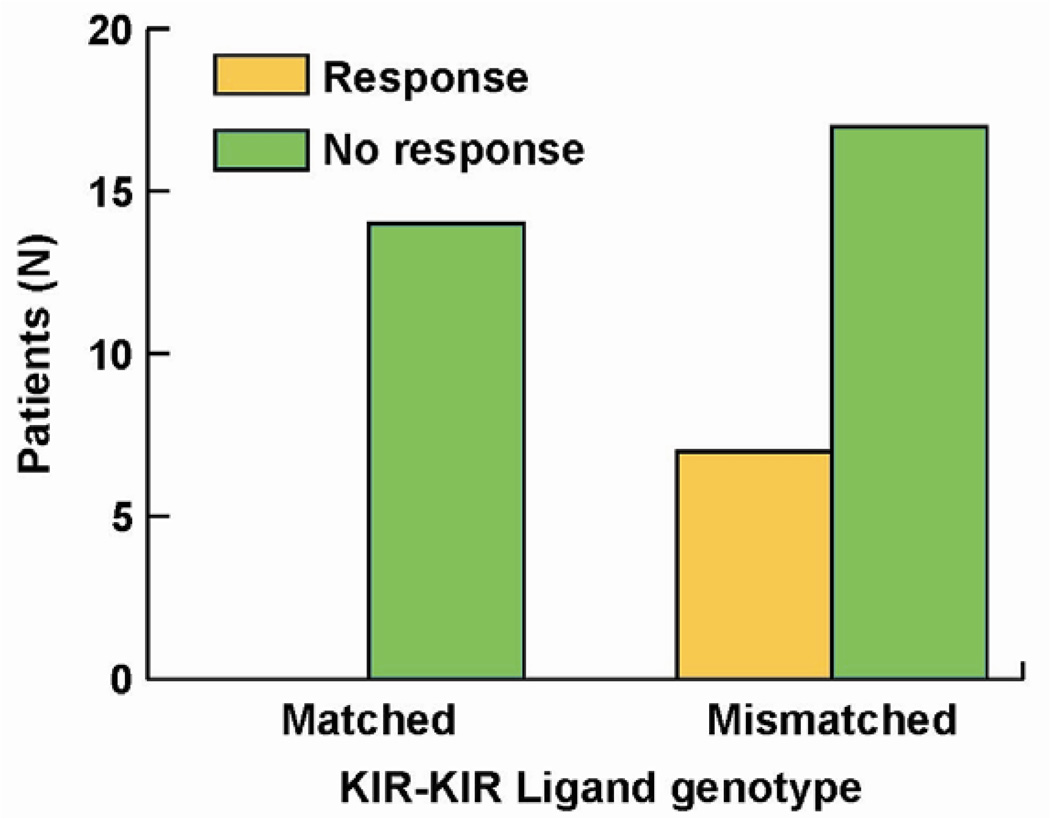
Importance of KIR/KIR-Ligand mismatch in clinical response to hu14.18-IL2. Of 38 individuals treated with hu14.18-IL2 in a phase II protocol [61] all of the 7 individuals that showed clinical benefit were from the group of 24 that were KIR/KIR-Ligand mismatched (*p* = 0.033) [84].

We performed KIR and KIR-L genotyping on the 38 pts. All 7 patients that showed improvement were in the cohort of patients that were KIR/KIR-L mismatched; none of the patients in the KIR/KIR-L matched group showed response or benefit (Figure 3*p* = 0.03). Even though our report [84] reflects a study of only 38 pts, this is the first demonstration (to our knowledge) that an individual’s KIR/KIR-L status may predict response to an immunotherapy that does not involve adoptive allogeneic cellular transfer or autologous HSCT. These results suggest that NK cells may have a significant role in the observed clinical anti-tumor activity.

In this same study we also evaluated FcR genotypes. We did find a trend (*p* = 0.06) for improved likelihood of benefit for thos patients that inherited the high affinity genotype (HH) for the FcRγ2a receptors on neutrophils and monocytes/macrophages [84]. This suggests that treatment with the hu14.18-IL2 may have been activating these FcRγ2a-bearing effector cells to mediate ADCC *in vivo*. It is notable that there was no correlation between response and FcRγ3a genotype [84]. A possible explanation for this is suggested by more recent pre-clinical studies with hu14.18-IL2 and ICs targeting other tumor types.

One such immunocytokine, DI-Leu16-IL2, targets CD20 using the de-immunized form of the murine mAb, Leu16, as the targeting agent. Mouse efficacy studies in SCID mice engrafted with disseminated lymphoma showed very high anti-tumor activity that was more than 50-fold more potent than that of rituximab, or the combination of rituximab and IL2 [85]. This activity was only partly attributable to the standard effector functions of ADCC and CDC because a de-glycosylated form of DI-Leu16-IL2 that lost both of these activities retained most of the anti-tumor activity. Since the primary effector cells in SCID mouse models are expected to be NK cells, and the de-glycosylated form of DI-Leu16-IL2 is not able to bind FcRs, we tested whether other mechanisms were responsible for activating NK killing. The first of these studies were performed with both hu14.18-IL2 and the anti-EpCAM IC, huKS-IL2, and showed that both agents induced target cell conjugation between NK cell lines lacking FcR but constitutively expressing CD25, and the tumor cell expressing the appropriate tumor target (GD2 or EPCAM, respectively) [86]. Furthermore, this conjugate formation led a polarization of CD25 to the immunological synapse and to enhanced target cell lysis [87]. We have named this novel effector function IFCC (immunocytokine-facilitated cellular cytotoxicity). Further studies are needed to assess whether other cell types expressing CD25, besides NK cells, are capable of this novel effector function but in the case of relapsed neuroblastoma, a strong correlation between KIR/KIR-L mismatch certainly implicates NK cells as the primary effectors in this clinical setting.

## 6. Augmenting Local Antitumor Activity by IT Injection of IC

**Figure 4:**
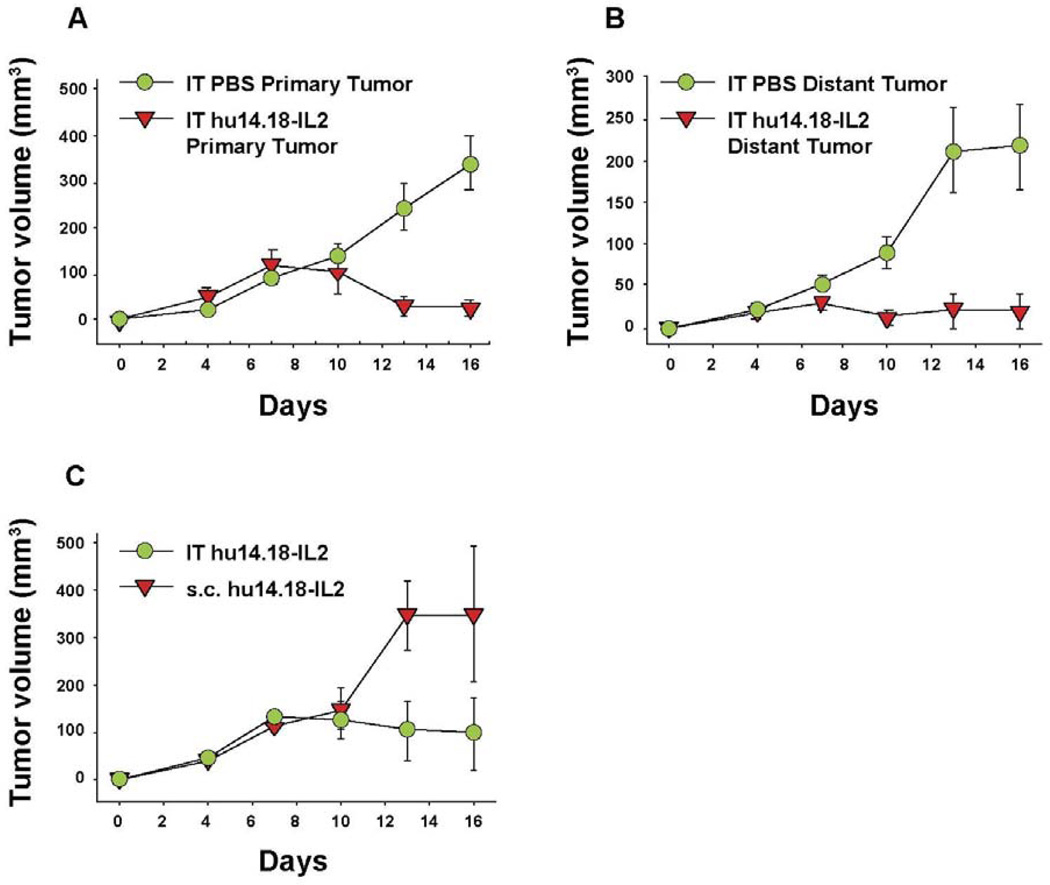
IT Immunocytokine effects agains primary (injected) and distant tumors. A/J mice (5 per group) received NXS2 (10^6^ s.c. on day 0) in the abdomen (primary tumor). A 2nd NXS2 injection (10^6^ s.c.) was placed on the flank on d4 (distant tumor). The primary tumor received 50 µL PBS or 15 µg hu14.18-IL2 IT on d7-11, while the distant tumor was not treated. **A**: Tumor volume of primary tumors (*p* = 0.001, day 16). Day 0 = implantation of the primary tumor. **B**: Tumor volume of distant tumors (*p* = 0.007, day 16). Day 0 = implantation of the distant tumor. **C**: A/J mice (3 per group) received 10^6^ NXS2 cells on d0 in the abdomen. Mice were treated with 15 µg hu14.18-IL2 IT or s.c. into the flank at a site away from the tumor (*p* = 0.04, days 13–16). (Adapted from Johnson et al, 2008 [91]).

**Figure 5:**
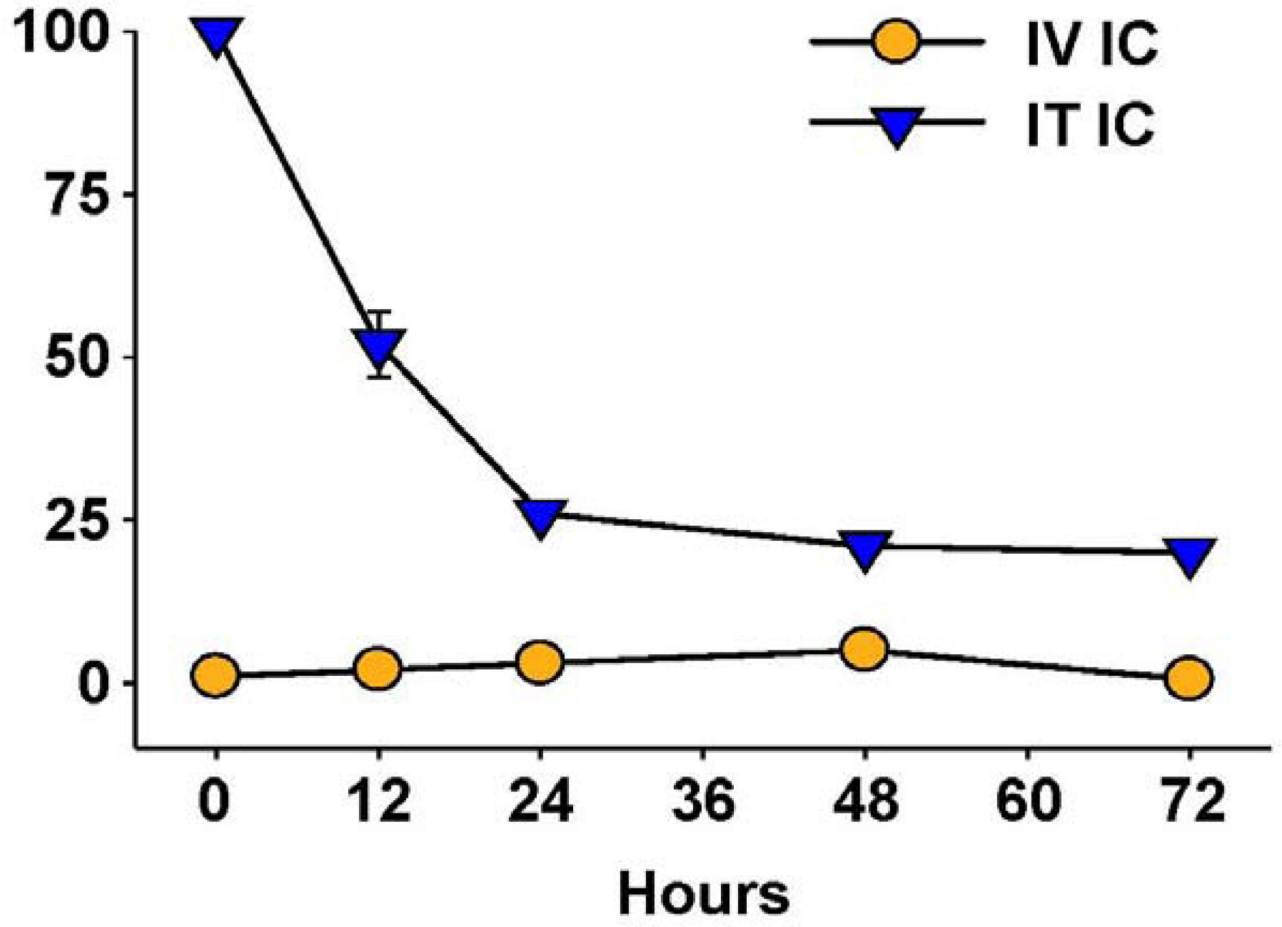
IT *vs*. IV delivery results in increased tumor retention of IC. CEA-transgenic C57BL/6 mice bearing d10 s.c. MC-38.CEA tumors were treated with ^111^In-GcT84.66-IL2 anti-CEA IC. Animals received 25 µg IV (5/grp) or 2.3 µg IT (2/grp). Tumors were harvested serially from the IV-treated mice and the % injected dose (shown on the Y-axis) determined by a γamma counter. IT-treated mice were imaged with a amma-camera, acquiring % injected dose by gating on the tumor. (Adapted from Johnson et al, 2008 [91]).

Hu14.18-IL2 administered IV has shown antitumor effects, particularly in mice with MRD, and preliminarily in patients with non-bulky disease [61] However, its effect against established “bulky” disease in both preclinical and clinical studies has been minimal or transient [39,61,88]. One potential explanation is that following IV injection of IC an inadequate amount of the IC is getting to measurable sites of disease to achieve a clinically significant effect. This is likely due to two major factors. The first is that more than 90% of ICs such as hu14.18-IL2 are lost on first pass clearance by the liver following IV injection [89]. The second is the interstitial pressure of solid tumors that makes it difficult for large proteins to enterfrom the circulation [90]. We tested this hypothesis by administering hu14.18-IL2 directly into subcutaneously (s.c.) established NXS2 tumors [91]. This IT IC (IT-IC) approach demonstrated significantly greater antitumor effects than IV administration of IC (IV-IC). Only 3 out of 17 mice treated with IV-IC cleared the tumor, while 12 of 17 mice treated with IT-IC cleared tumor (*p* = 0.002, IT *vs*. IV groups). IT treatment of a single s.c. tumor (Figure 4A) also prevented the growth of a separate s.c. tumor at a distant site (Figure 4B). Better antitumor effects were seen at distant tumor sites when the IC was injected into the primary tumor, than when the IC was injected s.c. into non-involved skin at an equidistant site (Figure 4C). This argues that injecting IC into the tumor induces a systemic immunologic effect, seen at distant tumor sites. There was also a memory response after IT-IC: most mice that became NXS2-free after IT-IC were able to reject subsequent re-challenges with NXS2 tumor cells, but didn’t reject an unrelated tumor (YAC-1). Preclinical studies have shown antitumor effects of IT injections of IL2, leading to clinical testing of IT injections of IL2, especially in melanoma [92,93]. IT administration of the IC was significantly more potent at tumor eradication than was IT administration of soluble IL2 (not shown) [91], or of IT injection of a non-specific IC, KS-IL2 (not shown) [91]. Far greater levels of IC are achieved and remain in the tumor site after 24 h following IT *vs.* IV delivery (25% *vs*. 4% of injected dose at the tumor, Figure 5) [91].

**Figure 6:**
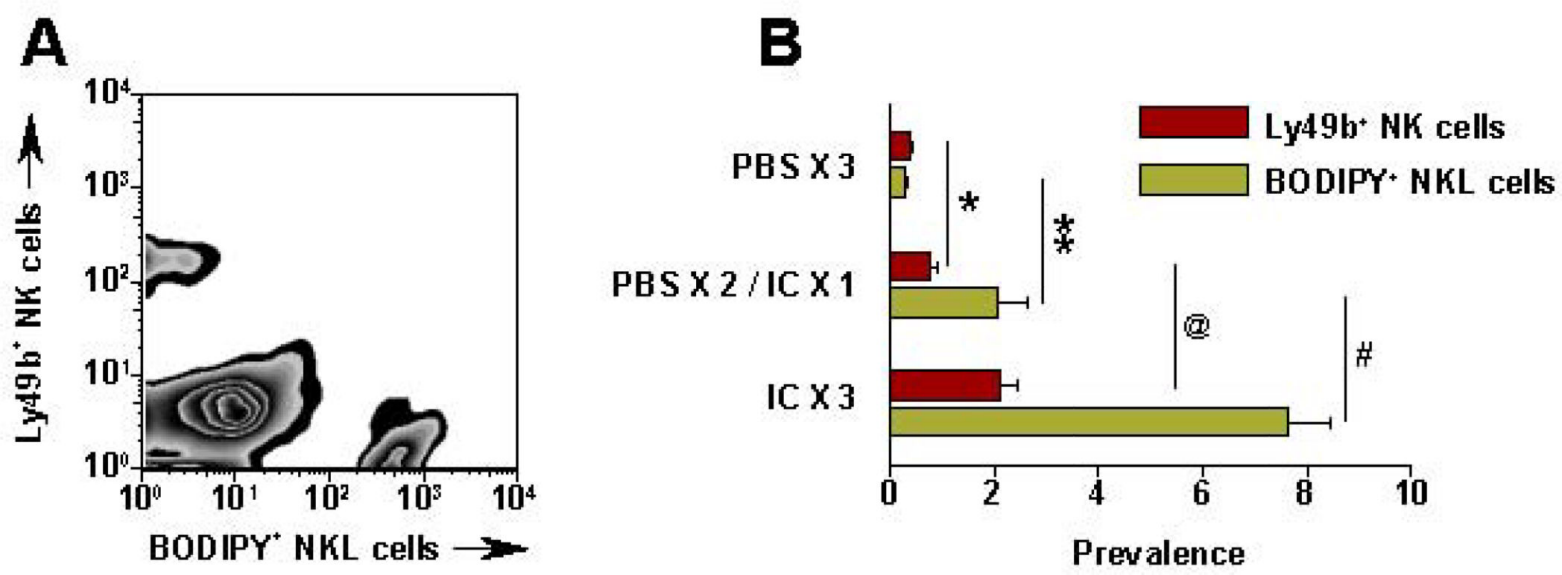
IT-IC facilitates migration of NK cells into tumor. SCID mice bearing s.c. M21 received IT-PBS (50 µl) or IT-IC (10 µg in 50 µL) on day 27–29. (PBSx3) = PBS on d 27, 28, 29; (PBS × 2/IC × 1) = PBS on d 27, 28, and IC on d 29; (IC × 3)= IC on d 27, 28, 29. On d 29, right after the last IT- PBS or IC, all mice received 5 × 10^6^ BODIPY-labeled NKL cells IV. 24 hr after the NKL cell injection, tumors were harvested, processed, stained and tested by flow for mouse Ly49b^+^ NK cells and BODIPY^+^ NKL cells. **A**: Representative pattern from a (IC × 3) mouse; numbers are for the 3 (ICx3) mice (Mean % ± SEM). **B**: Data for all groups (represents 2 independent experiments). * *p* = 0.06; ** *p* = 0.046; @ *p* = 0.02; # *p* = 0.007. (Adapted from Buhtoiarov et al 2011^87^).

Recent data show that IT administration of IC can also enhance localization of NK cells in the tumor site [87]. CFSE (Carboxyfluorescein succinimidyl ester, a fluorescent stain) labeled human NKL cells were injected IV (5 × 10^6^) into SCID mice bearing human GD2^+^ M21 melanoma tumors (~1 cm diameter) [87]. After 24 h, the tumors were harvested, disaggregated and evaluated by flow cytometry to determine what % of viable cells represented the CFSE-labeled NKL cells (Figure 6). In PBS treated NXS2 tumors, only 0.06% of the viable cells were CFSE labeled; in contrast, 24 h after IT-IC, 6.9% of the viable cells in the tumor were the CFSE labeled NKL cells [87]. IHC analyses of NXS2 tumors immediately following IT-IC show direct staining by IC and demonstrate NK infiltration 24 h later (data not shown).

**Figure 7:**
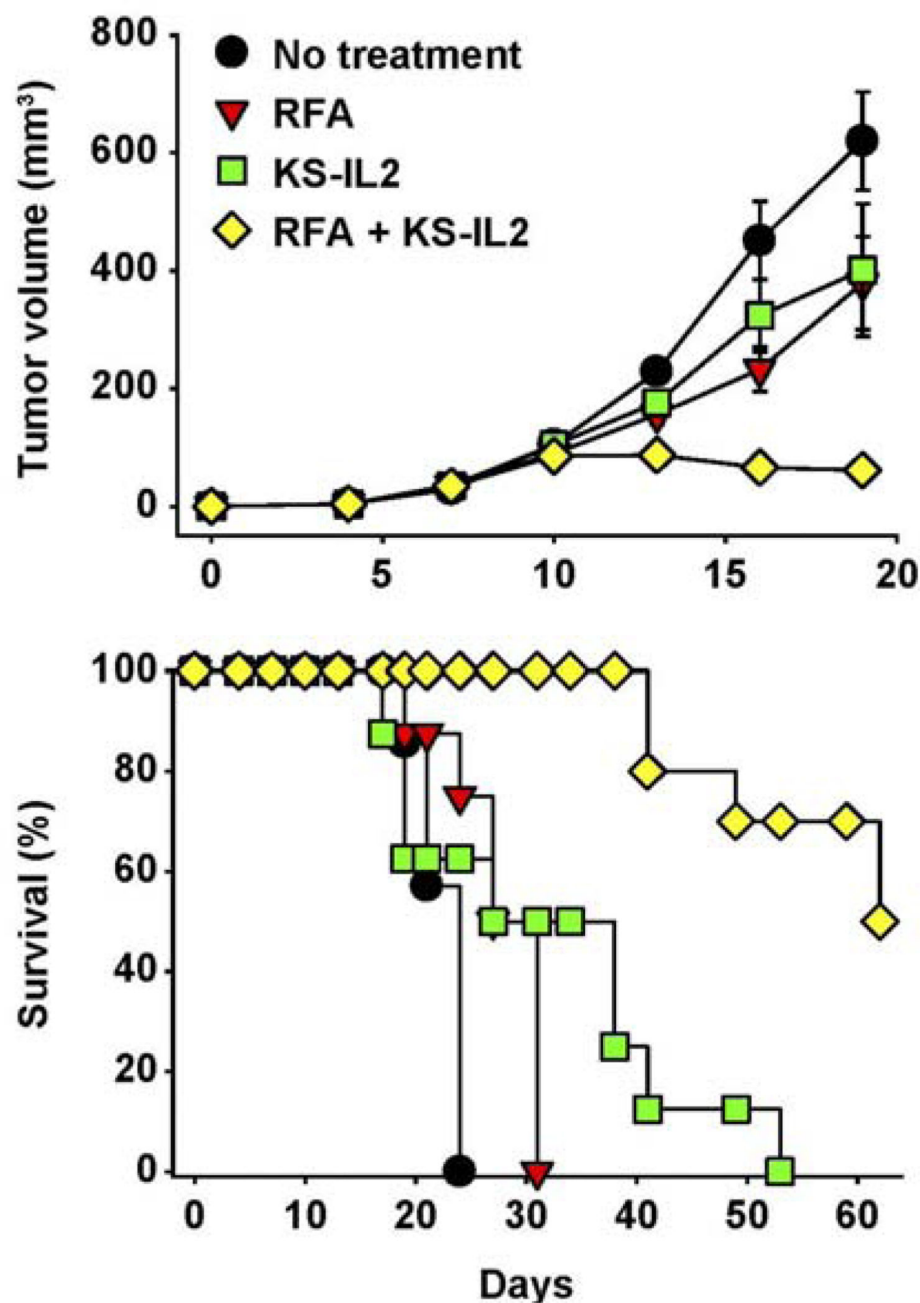
RFA and huKS-IL2 IC synergize in induction of antitumor effects. Balb/c mice (7–8 mice per group) received 5 × 10^5^ CT26-KS cells s.c. in the abdomen on d0. Mice were treated with partial RFA (25 seconds, d11), KS-IL2 IC (15 µg, d11–15), or both RFA and KS-IL2. Data shown are mean tumor volume ± SEM **(A)** or survival **(B)**. The results are representative of 3 experiments. (Adapted from Johnson et al, 2008 [91]).

As the localized injection of IC at a tumor site may not only induce ADCC and IFCC, but also serve as an endogenous, autologous tumor, vaccine, we have capitalized on the ability of apoptotic tumor cells to facilitate antigen uptake and presentation for vaccination purposes. In that regard, we have used radio-frequency ablation (RFA) to induce localized tumor cell damage (via thermal tissue injury) in combination with IT-IC treatment [94]. In many of these studies we investigated this in mouse models with the KS-IL2 IC directed against the Epithelial Cell adhesion molecule (EpCam). Dramatic synergy was seen when IT-IC was combined with RFA (Figure 7) [94]. Using a separate IC, synergy has been observed by combining IC treatment with localized radiotherapy, likely inducing similar synergistic mechanisms as the combination of IC + RFA in Figure 7. In this case, an IC targeting necrotic DNA and containing a form of IL2 specific for the high affinity IL2R was administered IV following radiation of s.c. Lewis Lung Carcinoma tumors. This resulted in the rapid regression of tumors in all mice that was preceded by cytotoxic T cell infiltration and followed by the ability of these mice to resist a subsequent tumor challenge (S.Gillies unpublished data).

These data support the following hypotheses: (a) IT-IC causes much higher and longer lasting levels of IC in the injected tumor than IV-IC; (b) Greater IC levels in the tumor enhance NK infiltration into the tumor (via FcRs and IL2Rs), leading to greater ADCC and greater tumor destruction, even of larger macroscopic lesions that are unresponsive to IV IC delivery; (c) Some of the IC injected IT circulates systemically (via lymphatics and blood vessels), enabling IC delivery to distant sites as effectively as when IC is given IV (possibly with a better PK profile); (d) The IC-facilitated response within the tumor may attract other effector cells (T cells and macrophages) to the site of necrotic tumor (or to draining lymph nodes), leading to T cell sensitization; (e) The vaccine-like effect resulting in tumor-specific T cell reactivity may impact on distant sites of micrometastatic disease (and prevent subsequent growth upon experimental tumor rechallenge); (f) Combining IC with other treatments that cause localized tumor damage (without local immune suppression), should synergistically augment the antitumor activity of the IC. All data in hand, which were generated with first-generation ICs, hu14.18- IL2 and huKS-IL2, are consistent with, but do not prove, all these hypotheses. Thus, while continued preclinical studies need to better understand the cellular and immune mechanisms of these antitumor effects, the antitumor efficacy of this approach and the relative ease for translating it to the clinical setting justify initial Phase I/II clinical testing of this IT-IC strategy, especially for melanoma.

## 7. Future Directions for IC Development and Treatment

The data, discussed above, have shown how more effective IT dosing can be for ICs with suboptimal pharmacokinetic properties. While the IT approach can be useful for cancers with easily accessible tumors, the treatment of disseminated, systemic disease might benefit from ICs that have been optimized for tumor targeting and reduced liver clearance. In this regard we reported technical approaches that greatly improved the PK properties of ICs – the genetic removal of the N-linked glycan in the CH2 domain of the H chain (that abrogates FcR binding) and alterations in the fusion junction between the H chain and IL2 that reduce intracellular proteolysis [95,96]. In the first case, blood levels are increased following IV administration, tumor targeting of radio-labeled IC is enhanced, and liver uptake is significantly reduced. In the second case, blood levels are also increased despite retaining FcR binding (and CDC activity), presumably due to more efficient re-cycling of the IC out of cells that have taken it up. In both cases, relatively low doses of the ICs were completely effective at causing the regression of s.c. colon carcinoma tumors in all mice, while the equivalent dose of the first-generation IC (huKS-IL2 in this case) showed only a short growth delay. This improved form of huKS-IL2 is not in clinical development so translation of these results to human therapy is currently not possible. Fortunately, the IC discussed above, DI-Leu16-IL2, was constructed using one of these optimization strategies and is in early clinical trials. In this case, the CH2 domain N-linked glycan was retained (preserving ADCC and CDC activity) but the H chain/IL2 fusion junction was modified for superior PK properties [85]. These changes have created an IC with very potent anti-lymphoma activity, even in the absence of T cells (since data was generated in SCID mice), and that has all effector functions (including IFCC). We are hopeful that DI-Leu16-IL2 studies in lymphoma patients will demonstrate T cell responses as well, since it is known that a high proportion of lymphoma patients have infiltrating T cells in the tumor microenvironment, and that they can be associated with better clinical outcome [97].

Another aspect of these ICs with improved PK properties is their potential for vascular toxicity. It is well known that IL2 toxicity, in large part, is related to Cmax levels in the blood and that this, in turn, is related to the activation of immune cells expressing the intermediate IL2 receptor, CD122 [98]. We have found two ways of greatly reducing the vascular toxicity of ICs based on this hypothesis. The first is through the genetic modification of IL2 at position D20 that interacts with the β chain of CD122 and thereby prevents activation of this receptor. This D20T mutation still allows the high affinity receptor to be activated when the α chain forms the trimeric complex with the β and γ chains [99]. This IC, called NHS-IL2LT (for low toxicity - also called Selectikine) preferentially activates cells expressing CD25, such as effector T cells, over CD122 expressing cells generally associated with vascular toxicity. Studies in both monkeys and cancer patients have shown a dramatic reduction in the IL2 toxicities normally seen with IL2-based ICs administered IV. The second approach to reducing vascular toxicity of IL2 based ICs is to administer them by the s.c. route instead of IV. This avoids the initially high Cmax obtained by IV administration and instead, the IC is taken up by the lymphatics and then slowly released into the bloodstream over a longer period of time. In this way cells expressing CD25 are preferentially activated without the overstimulation of cells expressing CD122. One possible advantage of this approach is that there is still some degree of CD122 activation that can up-regulate the expression of CD25 to increase the effector T cell pool, but still does so in a way that is well tolerated. The first IC that will be compared clinically using IV or s.c. dosing is the anti-CD20 IC, DI- Leu-16-IL2. Pre-clinical studies in monkeys have already confirmed the increased tolerability of s.c. dosing compared to the same doses given IV and studies in patients are underway. Since these studies will be done with patients with CD20-expressing cancers, such as non-Hodgkin’s Lymphoma (NHL), there may be an additional advantage of using s.c. dosing, since the drug is taken up by the compartment in which lymphoma generally resides and thereby enhances tumor targeting. For other cancer types that are not in the lymphatic system, further optimization of ICs may help increase the efficiency of s.c. uptake through the lymphatics and into the blood so that it can effectively target these tumors. We are currently developing such molecules that have longer circulating half lives, better uptake into the blood using s.c. injection and in this way benefit from this well tolerated and convenient route of administration. Further modifications also include modifications that alter the balance between the activation of CD25 and CD122-expressing cells. In this way we hope to find the optimal balance between the effector functions provided by the antibody component of the IC and the potent cytokine activity.

## Figures and Tables

**TABLE 1:** Efficacy of ch14.18-IL2 exceeds ch14.18 + IL2

Treatment	Tumor	Number of Tumor Foci
PBS IL2+ch14.18 **ch14.18- IL2	*NXS2 *NXS2 *NXS2	>250, >250, >250, >250, 240, 115 174, 134, 105, 102, 91, 83 0, 0, 0, 0, 0, 0,
ch14.18-IL2 PBS IL2+ch14.18 ch14.18-IL2	#B16 #B78 +B16 #B78 + B16 #B78 + B16	>500, >500, >500, >500, >500, 138, 97 >500, >500, >500, >500, >500, >500, >500,>500 >500, >500, >500, >500, 189, 179, 104 ##0, 0, 2, 7, 9, 12, 21, 43

* **Hepatic metastases** (mets) were induced with 10^6^ NXS2 cells IV into AJ mice. On day 1 mice received PBS, 10 mcg ch14.18 mAb + 30,000 IU IL2/d, or 10 mcg of ch14.18-IL2 daily×6d. Mets were scored for each of 6 mice on d21, and were less in the IC group

** than the other 2 groups (*p* < 0.001) [57];

# **Pulmonary mets** in C57Bl mice were induced by IV injection of 1x10^6^ B16 cells (GD2-) alone, or combined with 5x10^6^ B78 cells (GD2^+^). One week post-inoculation, 7d of PBS, 8 µg ch14.18 + 24,000 IU IL-2, or 8 µg ch14.18-IL2 was initiated, and mets were scored 4 wks later.

## Mice with mixed tumors treated with IC had fewer mets than all other groups (*p ≤* 0.002) [55].

**TABLE-2:** KIR/KIR-L Match-Mismatch Algorithm

Receptor	Ligand
KIR2DL1 (CD158a)	HLA-C2 (Lys80)
KIR2DL2/KIR2DL3 (CD158b)	HLA-C1 (Asp80)
KIR3DL1 (CD158e)	HLA-Bw4, HLA-A^Bw4^
